# Circular RNAs: Biogenesis, Biological Functions, and Roles in Myocardial Infarction

**DOI:** 10.3390/ijms24044233

**Published:** 2023-02-20

**Authors:** Jialei Li, Yu Han, Shuang Wang, Xiaolei Wu, Jimin Cao, Teng Sun

**Affiliations:** Key Laboratory of Cellular Physiology at Shanxi Medical University, Ministry of Education, Key Laboratory of Cellular Physiology of Shanxi Province, Department of Physiology, Shanxi Medical University, Taiyuan 030001, China

**Keywords:** circular RNAs, biogenesis, cyclization form, biological functions, myocardial infarction

## Abstract

Non-coding RNAs have been excavated as important cardiac function modulators and linked to heart diseases. Significant advances have been obtained in illuminating the effects of microRNAs and long non-coding RNAs. Nevertheless, the characteristics of circular RNAs are rarely mined. Circular RNAs (circRNAs) are widely believed to participate in cardiac pathologic processes, especially in myocardial infarction. In this review, we round up the biogenesis of circRNAs, briefly describe their biological functions, and summarize the latest literature on multifarious circRNAs related to new therapies and biomarkers for myocardial infarction.

## 1. Introduction

Myocardial infarction (MI) is an acute cardiovascular illness with an extremely high case fatality rate. Although there are existing clinical approaches, such as pharmacotherapy, coronary intervention reperfusion therapy, and stem cell transplantation [1,2], myocardial necrosis and remodeling after MI cause enormous damage to cardiac function, and there remains a considerable number of patients with undesirable outcomes [3].

MI is mainly caused by reduced coronary blood flow, which causes the damage and remodeling of the heart tissue. The critical pathogenesis is endothelial dysfunction induced by diverse etiologies, such as poor atherosclerotic plaque stability and various pathophysiological modifications such as atherosclerotic plaque rupture and erosion [4]. These lead to platelet aggregation and excitation, the excitation of endogenous and exogenous body coagulation mechanisms, the activation of a transition from fibrinogen to fibrin, and a combined range of actions, such as blood corpuscles [5]. The formation of thrombosis leads to the complete or partial obstruction of the coronary arteries in diseases and decreased myocardial perfusion, thus leading to MI [6]. The presence of thrombosis in an epicardial coronary artery, which supplies the myocardium, reduces or stops the blood flow in part of the heart, reduces myocardial perfusion, and leads to MI due to the apoptosis and necrosis of myocardial cells. After the onset of MI, the heart will also undergo a series of compensatory reactions, such as cardiac hypertrophy and fibrosis, which further lead to cardiac remodeling. In addition, the sustained excitability of the sympathetic nervous system (SNS) and the renin-angiotensin-aldosterone system (RAAS) after MI further worsens cardiac function.

In recent years, significant advances have been made in the study of microRNAs and long non-coding RNAs, while the serviceable impacts of circular RNAs have rarely been researched. Nevertheless, an increasing wealth of evidence supports the potential of the underlying mechanisms of circRNAs as new therapeutic targets and biomarkers for cardiovascular diseases, particularly MI. In this review, we summarize the present state of knowledge about circRNAs. We discuss their biological origins, mechanisms of cyclization, and potential as new biomarkers and therapeutic targets for heart disorders, emphasizing MI. 

## 2. Biogenesis and Features of Circular RNAs

In 1976, Sanger et al. [7] indicated that viroids are single-stranded, covalently closed RNA molecules with an unexceptionable self-complementarity and base-pair-making capacity. For the first time, circRNAs were discovered in viruses. Still, due to the limitations of biotechnology and human cognition, circRNAs were deemed to be the by-products of abnormal splicing and were ignored. Soon after, in 1979, Hsu and Coca-Prados at Rockefeller University first detected the presence of circRNAs in human HeLa cells through electron microscopy [8]. In 1980, circRNAs were found in the mitochondrial genome of yeast [9]. In the following period, circRNAs were occasionally found in mammals [10,11]. Specific circRNAs derived from the *sex determination region Y (SRY)* gene were found and considered to have a possible function in mouse testes [12]. In 2012, Danan et al. [13] found that circRNAs were abundant in archaea and had specific biological functions. The rapid development of RNA sequencing tools has led to the verification of large quantities and varieties of circRNAs. Jeck et al., the authors of [14], identified at least 25,000 circRNAs in human fibroblasts. This suggests that circRNAs are also associated with various functions of the heart. Thus far, researchers have discovered many circRNAs in eukaryotes, and they have determined that these RNAs have tissue-specific, cell-specific, and developmental-stage-specific manifestations [14,15,16,17,18]. These numerous scientific discoveries have rendered circRNAs a new star in non-coding RNA molecules, following microRNAs and long-stranded non-coding RNAs.

CircRNAs are diffusely present in multifarious human cells, peripheral blood, saliva, and other body fluids. In some cases, they are significantly more abundant than the related linear RNAs [19]. In 2021, it was reported that the expression of a cardiac-necroptosis-associated circRNA (CNEACR) was downregulated in myocardial infarction [20], being closely related to the progression of myocardial infarction. Subsequently, in 2022, another study highlighted the selective expression of circSamd4 in fetal and neonatal cardiomyocytes [21]. It also plays a crucial role in myocardial infarction. This is indicative of the tissue specificity of circular RNAs. At the same time, researchers have demonstrated that circRNAs abound in tissues that divide slowly, such as nerve cells or other highly differentiated cells, and are low in tissues that divide rapidly, such as cancer cells [22].

CircRNAs are abundant, stable, widely distributed, and specifically expressed, and so are a potential ideal candidate for non-invasive biomarkers and a regulatory target for MI. The latest study noted that a total of 3,862 differentially expressed circRNAs were identified by the bioinformatics and high-throughput RNA-seq analysis of RNA from the peripheral blood. By calculating and creating the receiver operating characteristic (ROC) curve, the authors noted that *circTMEM165*, *circUBAC2*, *circZNF609*, *circANKRD12*, and *circSLC8A1* have a high sensitivity and specificity in the determination of myocardial infarction [23].

## 3. Cyclization Form of Circular RNAs

**Figure 1:**
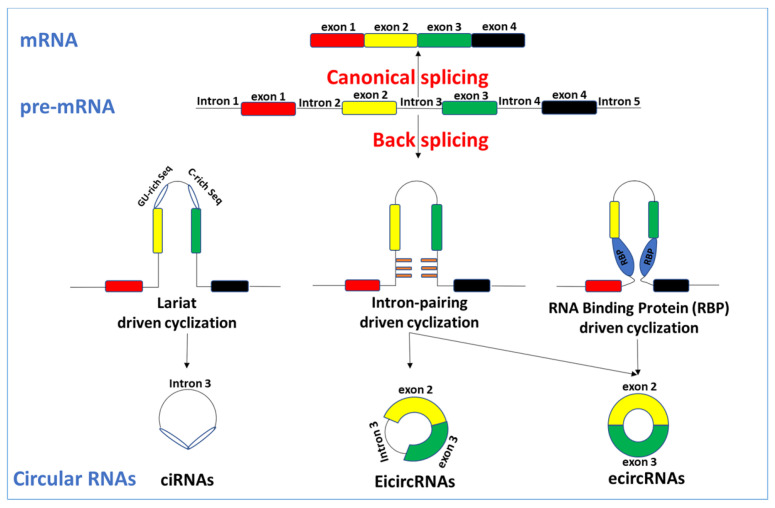
The cyclization forms of circular RNAs. A gene produces linear messenger and circular RNAs through two different splicing methods. The cyclization forms of circular RNAs are described in detail in the text. (i). Lariat-driven cyclization forms ciRNA. (ii). Intron-pairing-driven cyclization forms EicircRNA. (iii). RNA-binding-protein (RBP)-driven cyclization forms ciRNA. In this diagram of the cyclization forms of circular RNAs, exon 3 is associated with the upstream exon 2 rather than exon 4, as observed in linear splicing.

The mechanisms of RNAs are related to pre-mRNA splicing [15,24]. In eukaryotes, pre-mRNAs undergo a process called “canonical splicing”. The main splicing form of “typical splicing” involves the splicing of introns of pre-mRNAs by spliceosomes and ligation of exons into a linear RNA transcriptome, known as mRNA. Parallel with linear mRNAs, pre-mRNAs are also precursors of circRNAs. The pre-mRNAs undergo a process called “back splicing.” “Back splicing” is opposed to “canonical splicing,” which forms linear mRNAs. “Back splicing” is a unique splicing mechanism for forming circRNAs. Based on the “back splicing” approach, circRNAs lack the typical 5′ caps and 3′ poly (A) tail and form a stable, covalent, closed-loop frame. There are three primary cyclization forms of circular RNAs (Figure 1).

### 3.1. Lariat-Driven Cyclization

Typically, precursor RNA removes the introns during processing, and the introns are degraded by exonuclease after a debranching reaction. However, when pre-RNA has a sequence rich in 7nt guanine (G) and uracil (U) (a Gu-rich sequence) near one exon and an 11nt cytosine (C)-rich sequence (C-rich sequence) near another exon, during the splice reaction that functions to produce the intron noose the introns can avoid debranching and cyclize to form firm circRNAs [25].

### 3.2. Intron-Pairing-Driven Cyclization

The introns upstream and downstream of exons contain reverse complementary cis-acting elements, such as the ALU sequence, which are paired directly through base complementation, so that the splicing sites are spatially close to each other, forming exon–intron circRNA without intron removal or exon circRNA with intron removal. The diversity of the ALU sequences renders the pairing selective and competitive, generating multiple carcasses in the same gene [24].

### 3.3. RNA-Binding-Protein (RBP)-Driven Cyclization

The pre-mRNAs can be driven to circRNAs by RNA-binding protein (RBP). RBP can recognize and anchor specific gene sequences in introns and create splicing sites at both ends of exons that are close to each other through protein interaction or dimer formation, forming covalent links between the splicing acceptor and splicing donor, and, finally, cyclization, leading to the formation of cercariae [26]. 

Based on their molecular mechanisms of biogenesis, circRNAs are usually categorized into three types: (1) intronic circRNAs (ciRNAs), which refer to those circRNAs composed only of introns, which are located in the nucleus; (2) exonic circRNAs (EcircRNAs), which refer to the circRNAs composed only of exons, mainly existing in the cytoplasm; and (3) exon–intron circRNAs (EiciRNAs), which refer to the circRNAs that retain introns between their exons, principally existing in the nucleus. 

The parental gene’s location can be used to separate circRNAs into intragenic circRNAs and intergenic circRNAs [27]. Each EcircRNA, EiciRNA, and circRNA molecule is derived from the splicing of different exons and/or introns within the same parent gene and, therefore, belongs to the intra-gene circRNA. In addition, circRNAs derived from genomic intervals between different genes are called inter-gene circRNAs [24].

## 4. Biological Functions of CircRNAs 

### 4.1. CircRNAs as miRNA Sponges

Recently, the regulatory connection between circRNAs and microRNAs has drawn the attention of many researchers [28]. The interactive regulation of circRNAs and microRNAs is a focus of current research. 

The sponging of miRNAs refers to the fact that microRNAs are connected to the 3’ untranslated region of mRNA, thereby inhibiting the translation of mRNA. CircRNAs comprise miRNA response elements (MREs), which are miRNA-integrating sites. The sponge adsorption of miRNAs by circRNAs is mainly accomplished through the MREs. CircRNAs have multiple miRNA-binding sites, and single circRNAs have many good or excellent miRNA-binding sites. Numerous discoveries have shown that circRNAs have the function of a miRNA sponge. As molecular sponges of miRNAs, circRNAs play a biological role in regulating miRNAs. Consistent with the miRNA sponge, the molecular sponge role of circRNAs means that it can bind to miRNA as a binding site of competitive endogenous RNA and restrain the biological viability of miRNA, thus indirectly managing the manifestation of downstream target molecules. The expression level of the corresponding target gene increases when the circRNAs are overexpressed, while the expression level of the corresponding target gene decreases when they are inhibited. 

### 4.2. CircRNAs as Protein Sponges

CircRNAs occupy not only miRNA-binding elements but also bonding sites for protein. Research has indicated that partial circRNAs have protein-bonding sites and serve as protein sponges. CircRNA–protein interactions have been shown to impact protein expression, biogenesis, and pathophysiological progress [29,30]. Most circRNAs are derived from genes that encode proteins, and their frames can include exon sequences. CircRNAs binding to their target proteins will cause the functional suppression of these proteins [31].

### 4.3. CircRNAs Directly Translate Proteins 

Translating a gene into a protein requires a translation initiation complex. The formation of this complex requires the recognition of the 5’ cap structure of mRNAs [32]. Interestingly, studies have shown that circRNAs can be translated into proteins.

Research indicates that circRNAs with internal ribosome entry site (IRES) elements can initiate protein translation. The first circRNA identified as a protein translator was a single-stranded circRNA found in the hepatitis C virus that produces a protein with 122 amino acids [33]. Yang et al. [34] identified several peptides translated by circRNAs in human fibroblasts and found that when adenosine is methylated, the common motif modified by 6-methyladenine upstream of the start codon promotes the translation ability of circRNAs. Other studies have shown that *CIRCZNF609* can bind to the polyribosomes in mouse and human myoblast cells and perform protein translation in a splice-dependent and cap-dependent manner [35]. *Circ-FBXW7* can be translated into the protein F-Box and WD repeat domain-containing 7 (FBXW7)-185aa, but the roles of this protein remain largely undiscovered [36]. The circRNAs of the virusoid include internal ribosome entry sites and show an emerging coding ability. 

Although circRNA is understood to have a specific translation capacity, it has been demonstrated that the translation productivity could be higher owing to the influence of its unique ring structure. The functions of circRNA translation products are still unclear and warrant further investigation.

### 4.4. CircRNAs Directly Affect Gene Expression 

CircRNAs are released into the cytoplasm and extracellularly, giving them the capacity to act as biomarkers. However, to a lesser extent, sectional circRNAs exist in the nucleus. These nuclear circRNAs can respond to RNA polymerase Ⅱ in the promoter region of the host genes and modulate transcription [37].

CircRNAs participate firsthand in the modulation of gene regulation by adjusting the transcription of linear RNA. When pre-mRNA contains the translation initiation site, if cyclization and non-linear splicing occur, the transcribed mRNA will be reduced, reducing the number of downstream proteins for translation. This effect is called the “mRNA trap” [38]. 

### 4.5. CircRNAs Regulate mRNA Stability

A few circRNAs can similarly regulate mRNA stability. Hansen et al. [39] found that the circular RNA derived from the *cerebellar-degeneration-related protein 1 (CDR1)* gene can form a double-stranded structure with *CDR1* mRNA to enhance its stability. The *Circ-Ras-GEF domain family member 1B (Ras-GEF1B)* in murine macrophages enhances the strength of *intercellular cell adhesion molecule-1 (ICAM-1)* mRNA, causing the increased expression of ICAM-1 in the lipopolysaccharide (LPS)/toll-like receptor 4 (TLR4) inflammatory signaling pathway [40]. *CircFndc3b* stabilizes mRNA fused in the sarcoma (FUS), because circFndc3b is mainly gathered in the cytoplasm [41].

### 4.6. CircRNAs Regulate DNA Methylation 

The latest controlled mechanism of circRNAs to have been discovered is that circRNA adjusts gene regulation by influencing the DNA methylation of downstream genes. For example, *circRNA ACR* activates PTEN-induced putative kinase 1 (Pink1) expression by directly binding to DNA Methyltransferase 3 Beta (Dnmt3B) and deterring the Dnmt3B-mediated DNA methylation of the Pink1 promoter [42].

### 4.7. CircRNAs Act as a Retrotransposon to Mediate Pseudo-Genetic Production

A recent study found that circRNA antilock can form pseudogenes [43]. The exon sequence of pseudogenes derived from linear mRNA is consistent with that of the normal gene. Still, the exon connection sequence of pseudogenes derived from circRNA is opposite to that of the common gene. This indicates that circRNAs can serve as reverse transposons to alter the structure of the genome and adjust gene expression.

In addition to the functions mentioned above, circRNAs are likewise involved in the modulation of transcriptional, post-transcriptional, and translation processes such as RNA processing, genome rearrangement, chromosome modification, and X chromosome silencing [44]. It can be seen that circRNAs are powerful and worthy of further study. 

## 5. Conclusions on the Specific Functions of Circular RNAs in Myocardial Infarction

**Figure 2:**
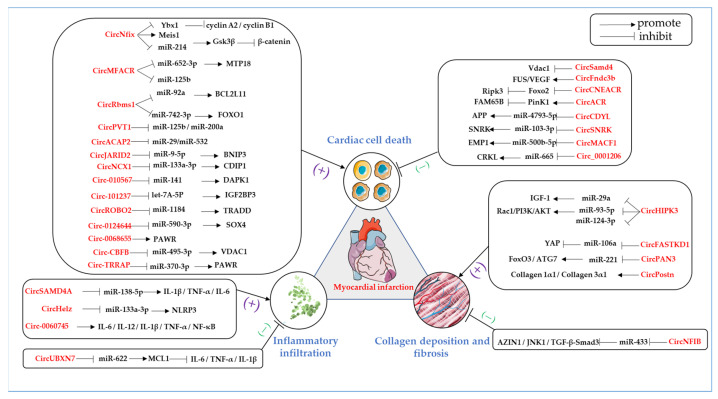
The role of circular RNAs in the pathogenesis of myocardial infarction, including cardiac cell death, inflammatory infiltration, and collagen deposition and fibrosis. All circular RNAs are labeled in red. They are described in detail in the text. The arrows in the figure represent the role of promotion. The T bars indicate the effect of inhibition [20,21,41,42,45,46,47,48,49,50,51,52,53,54,55,56,57,58,59,60,61,62,63,64,65,66,67,68,70,71,72,73,74,75,76,77].

Circular RNAs are distributed extensively throughout the cardiovascular system and vary dramatically in their expression levels in the context of different cardiac disorders, such as acute MI, angina pectoris, and myocarditis, and function as regulators of heart development, cardiac function, and stress response. Plenty of research has indicated that the critical features of circular RNAs are related to MI [45,46,47,48,49,50,51,52,53,54,55,56,57,58,59,60,61,62,63,64,65,66,67,68,69,70,71,72,73,74,75,76,77]. These circRNAs with altered levels have been found to regulate cardiac cell death after the onset of MI, such as apoptosis, autophagy, necrosis, and inflammatory infiltration, and also regulate myocardial collagen deposition and fibrosis during the healing stage of MI. Here, we seek to describe the circular RNAs that are closely related to MI in terms of the promotion or suppression of the pathology of MI (Table 1 and Figure 2).

### 5.1. Role of Circular RNAs in Cardiac Cell Death and MI 

Since adult mammalian cardiomyocytes are terminally differentiated cells with a very limited proliferative capacity, cardiomyocyte death during MI can result in the permanent loss of the cardiac functional unit. We attempted to summarize the role of circRNAs in promoting MI by regulating apoptosis, autophagy, and necrosis to provide a more comprehensive reference base for circRNAs involved in regulating cardiomyocyte death during MI.

#### 5.1.1. CircRNAs Promote Cardiac Cell Death in MI

- *CircNfix*

*CircNfix* has been demonstrated to be deeply involved in the MI regulation network. Huang et al. [45] conducted the first comprehensive analysis of the relationship between the super-enhancers and circRNA networks in the heart. They identified conserved cardiac circRNAs regulated by the super-enhancer Nfix, namely *CircNfix*. They found that the downregulation of *circNfix* can induce cardiomyocyte proliferation, promote angiogenesis, and inhibit cardiac apoptosis. Further research has shown that *circNfix* promotes myocardial infarction through the following mechanisms. *CircNfix* strengthens the mutual effects between Y-box-binding protein 1 (Ybx1) and NEDD4-like E3 ubiquitin-protein ligase (Nedd4L), facilitates Ybx1 ubiquitin-dependent degradation, and suppresses the expression of cyclin A2 and cyclin B1. *CircNfix* inhibits cardiomyocyte proliferation and angiogenesis after MI by promoting Ybx1 degradation mediated by ubiquitination, which promotes cardiomyocyte apoptosis and impairs cardiac function. *CircNfix*-associated super-enhancers were found to promote *circNfix* expression by promoting angiogenesis through *myeloid Homeobox 1 (Meis1)*, a prominent transcription factor that controls cardiac cell cycle arrest. Meis1 knockdown significantly represses glycogen synthase kinase 3β (Gsk3β) expression. Gsk3β is well known to inhibit cardiomyocyte proliferation via the degradation of β-catenin. In addition, *circNfix* may function as a *miR-214* sponge to upregulate Gsk3β expression and dampen β-catenin activity. *CircNfix* impairs cardiomyocyte proliferation and the Meis1/Gsk3β pathways. Interestingly, the expression levels of some circRNAs are inversely regulated by their roles, which appear to be compensatory transformations. A study conducted by Cui et al. [46] showed that *circNfix* promotes cardiomyocyte apoptosis, but the expression of *circNfix* exhibits a downward trend in apoptotic cardiomyocytes and ischemic hearts. Whether upregulated or downregulated, *circNfix* is a pro-apoptotic factor in MI and a potential biomarker and therapeutic target of MI.

- *CircACAP2*

*CircRNA ACAP2* was found to be increased in rat hearts subjected to MI and can mediate cardiac apoptosis by sponging *miR-29*, thereby promoting MI [47]. In 2021, it was reported that ArfGAP with coiled-coil, ankyrin repeat, and PH domains 2 (ACAP2) and mature *miR-532*, rather than premature *miR-532*, are significantly increased in plasma from patients with MI [48]. In vitro experiments similarly showed that the expression levels of ACAP2 and mature *miR-532* are increased in AC16 cells under hypoxia conditions. Nevertheless, ACAP2 and mature *miR-532* are significantly and negatively related. The enforced expression of ACAP2 promotes hypoxia-induced apoptosis by interrupting the maturation of *miR-532*.

- *CircMFACR*

When exposed to hypoxia/reoxygenation (H/R) experimental conditions, circRNAs, named *mitochondrial-fission-and apoptosis-related circRNA (MFACR)*, are substantially increased [50]. *MFACR* promotes MI progression by sponging *miR-652-3p* and favors the upregulation of the mitochondrial 18 kDa (MTP18) protein, which positively regulates mitochondrial fission and apoptosis in cardiomyocytes [78]. Another study revealed that the expression level of *MFACR* shows an upward trend in plasma samples from MI patients, MI animal models, and AC16 cells under hypoxia. At the same time, *miR-125b* is remarkably downregulated [49]. The authors further found that the overexpression of *MFACR* reduces the *miR-125b* levels, arising from the increased methylation of the miR-125b gene, thereby exacerbating apoptosis and deteriorating MI. The many recent findings on *MFACR* mark a new chapter in the study of circRNAs regulating mitochondrial dynamics and cardiomyocyte apoptosis.

- *CircPVT1*

The *circRNA PVT1 (circPVT1)* is highly expressed in MI tissues and H/R-treated cardiomyocytes [51]. *CircPVT1* functions as a competing endogenous RNA by competitively combining with both *miR-125b* and *miR-200a*. *CircPVT1* plays a protective role in myocardium derived from MI and H/R damage by attenuating *miR-125b*- and *miR-200a*-mediated apoptotic signaling pathways, including the p53/TNF-receptor-associated factor 6 (TRAF6), Sirtuin 7 (SIRT7), Kelch-like ECH-associated protein 1 (KEAP1)/nuclear factor erythroid-2-related factor 2 (Nrf2), and programmed cell death 4 (PDCD4) pathways.

- *CircNCX1*

The circRNA transcribed from the *sodium/calcium exchanger 1 (NCX1)* gene is *circNCX1*. *CircNCX1* is significantly upregulated and exposed to hypoxia/reoxygenation (H/R) and promotes cardiac apoptosis [52]. Furthermore, *CircNCX1* has been demonstrated to bind and inactivate *miR-133a-3p*, which aggravates ischemic myocardial injury induced by the pro-apoptotic protein cell-death-inducing P53 target 1 (CDIP1). The *CircNCX1*/*miR-133a-3p*/CDIP1 axis could provide a potential approach to facilitate treatment decisions for ischemic heart disease.

- *CircJARID2*

It has been reported that hypoxia amplifies the upregulation of *circJARID2* expression in H9C2 cells. The knockdown of *circJARID2* inhibits hypoxia-treated apoptotic cell death by releasing *miR-9-5p* to downregulate BCL2 interacting protein 3 (BNIP3) expression [53]. In conclusion, *circJARID2* promotes hypoxia-induced cardiomyocyte injury during the pathogenesis of MI.

- *CircROBO2*

*CircROBO2* is markedly upregulated in myocardial samples in response to ischemic damage. *CircROBO2* enhances the expression of TNFRSF1A-associated death domain protein (TRADD) by sponging *miR-1184*, which promotes apoptotic cell death and exacerbates cardiac dysfunction after myocardial infarction [54]. This study revealed a novel regulation model of AMI consisting of *CircROBO2*, *miR-1184*, and TRADD.

- *CircRbms1*

The *CircRbms1* expression levels are upregulated in I/R mice and H_2_O_2_-treated H9c2 cells. The upregulated *circRbms1* induces cardiomyocyte apoptosis and ROS release via the sponging of *miR-92a*. The authors showed that after knocking down *circRbms1*, *miR-92a* expression was upregulated, and the protein level of BCL2L11 in the heart tissue of the I/R mice was significantly reduced, alleviating cardiomyocyte apoptosis and cardiac function [55]. The *circRbms1*/*miR-92a*/BCL2L11 axis may be a potential biomarker for AMI therapy. In addition, other studies have confirmed the results of this work. *CircRbms1* is upregulated in MI mouse cardiac tissue and hypoxia-induced cardiomyocytes. The overexpression of *circRbms1* further aggravates hypoxia-induced cardiomyocyte damage. Studies have shown that *circRbms1*, by sponging *miR-742-3p*, promotes hypoxia-induced cardiomyocyte damage. FOXO1 is a target of *miR-742-3p*, and its expression is positively regulated by circRbms1 [56]. The *CircRbms1*/*miR-742-3p*/FOXO1 axis is also a critical pathway for the regulation of cardiomyocyte death.

- *CircCBFB*

The study found that the expression of *circ-CBFB* was upregulated in a hypoxia/reoxygenation (H/R) injury cardiomyocyte model. *Circ-CBFB* inversely regulates *miR-495-3p* expression by acting as a competing endogenous RNA. Interestingly, the study identified that voltage-dependent anion channel 1(VDAC1) as a functional target of *miR-495-3p* that is positively regulated by *circ-CBFB*. The absence of *circ-CBFB* significantly inhibited H/R-induced oxidative stress in the cardiomyocytes, enhanced cell viability, and inhibited apoptosis [57]. *Circ-CBFB*/*miR-495-3p*/VDAC1 is an innovative axis in H/R challenge cardiomyocyte injury, providing new ideas for the management of acute myocardial infarction.

- *CircTRRAP*

The expression of *circTRRAP* was significantly increased after hypoxia treatment in AC16 cells. *Circ-TRRAP* can target *miR-370-3p*. PAWR is a target of *miR-370-3p*, which is regulated by the *circ-TRRAP*/*miR-370-3p* axis. The protective effect of *miR-370-3p* is achieved by downregulating PAWR expression in hypoxia-treated AC16 cells. The downregulation of *circ-TRRA* inhibits cardiomyocyte apoptosis, inflammation, and oxidative stress [58].

In addition to the circRNAs mentioned above, many unnamed circRNAs promote apoptosis through the sponging of miRNAs.

Research has demonstrated that *circRNA 010567* is increased and miR-141 is decreased in H9C2 cells treated with hypoxia. *CircRNA 010567* increased the expression of DAPK1 by sponging *miR-141*, thus inhibiting cell proliferation, promoting apoptosis, and aggravating hypoxia-induced cardiac injury [59]. Furthermore, *circRNA_101237* is a novel circRNA produced by exons 10 to 12 of the *cyclin-dependent kinase 8 (CDK8)* gene. Anoxia/reoxygenation (A/R) treatment led to a gradual time-dependent increase in the expression level of *circRNA_101237*. *CircRNA_101237* induces the upregulation of insulin-like growth factor 2 MRNA-binding protein 3 (IGF2BP3) by sponging *let-7A-5P*, which activates autophagy to mediate cardiomyocyte apoptosis [60]. *Circ_0124644* is highly expressed in acute MI patients and hypoxia-induced AC16 cells. *Circ_0124644* acts as a ceRNA, sponging *miR-590-3p* to accelerate cardiomyocyte damage. It is also worth noting the opposite effect of *miR-590-3p*, which alleviates cardiomyocyte damage by targeting SRY-box transcription factor 4 (SOX4). The *Circ_0124644*/*miR-590-3p*/SOX4 axis has been demonstrated to be a novel pathway that regulates AMI onset and progression [61]. *Circ_0068655* is upregulated in AMI myocardial tissue and cardiomyocytes induced by hypoxia. *Circ_0068655* is pivotal in accelerating cardiac apoptosis by upregulating the PRKC apoptosis regulator (PAWR) [62]. These studies suggest that a large group of circRNAs contribute to MI regulation, and further exploration is required.

#### 5.1.2. CircRNAs Suppress Cardiac Cell Death in MI

- *CircCNEACR*

*Cardiac-necroptosis-associated circRNA (CNEACR)* is decreased in cardiomyocytes exposed to H/R and the hearts of mice in response to I/R. Following overexpression, CNEACR immediately combines with histone deacetylase 7 (HDAC7) in the cytoplasm, disturbing its entry into the nucleus, which causes the attenuation of the HDAC7-dependent suppression of forkhead box protein A2 (Foxo2) transcription. The CNEACR-mediated upregulation of Foxo2 inhibits the receptor-interacting-serine/threonine-kinase-3 (Ripk3)-dependent programmed necrosis of cardiomyocytes. The *CNEACR*/HDAC7/Foxo2/RIPK3 axis has a decreased MI area and ameliorated cardiac function [20].

- *CircSamd4*

*CircSamd4* is a circular RNA that is located in the mitochondria. Studies have highlighted the selective expression of *circSamd4* in fetal and neonatal cardiomyocytes. The overexpression of *circSamd4* can induce myocardial cell proliferation after myocardial infarction and inhibit cardiomyocyte apoptosis, reducing myocardial fibrosis. In light of this, the authors noted that *circSamd4* downregulates Vdac1 expression and prevents the mitochondrial permeability transition pore (mPTP) from opening, enabling it to function [21].

- *CircACR*

In response to I/R injury, a type of *autophagy-related circular RNA (ACR)* is significantly reduced [42]. The forced expression of ACR protects the heart from I/R injury, including a reduction in the myocardial area and improvement in cardiac function by inhibiting autophagic cell death. Pink1 regulates the core process of autophagy, in which a family with the sequence similarity 65 member B (FAM65B) has been identified as the target of Pink1. *ACR* activates Pink1 expression by immediately combining with DNA methyltransferase 3 beta (Dnmt3B), which obstructs the Dnmt3B-mediated DNA methylation of the Pink1 promoter. The discovery of ACR has revealed a new role of circRNAs in adjusting autophagy, and the *ACR*/PinK1/FAM65B axis may offer a prospective therapeutic target for the treatment of cardiovascular diseases.

- *CircCDYL*

*CircCDYL* is a new regulator of myocardial generation after MI, which may improve cardiac function. *CircCDYL* is significantly downregulated in cardiac tissue and hypoxic cardiomyocytes. The overexpression of *circCDYL* can increase the expression level of the amyloid precursor protein by sponging *miR-4793-5p*, thus inducing myocardial regeneration after MI [72]. This suggests that *circCDYL* is likely to be a valuable therapeutic target in improving the prognosis of MI.

- *CircFndc3b*

*CircFndc3b* is dramatically downregulated in the cardiac tissues of patients with ischemic cardiomyopathy and mice with MI. Recently, Garikipati et al. [41] demonstrated that the upregulation of *circFndc3b* provides protection against cardiac apoptosis and enhances angiogenesis and left ventricular function after MI via the FUS/vascular endothelial growth factor (VEGF) signaling axis.

- *CircSNRK*

*CircRNA SNRK* is downregulated in response to MI. Upon MI, the enforced expression of *circSNRK* reduces cardiomyocyte apoptosis, promotes cardiomyocyte proliferation, enhances angiogenesis, and improves cardiac function. In addition, *circSNRK* plays a cardioprotective role by absorbing *miR-103-3p* and increasing the level of SNF-related kinase (SNRK), which can combine with GSK3β to modulate its phosphorylated activity [73].

- *CircMACF1*

*CircMACF1*, as a sponge of *miR-500b-5p*, upregulates the expression of epithelial membrane protein 1 (EMP1), which effectively restores cardiomyocyte apoptosis in MI [74]. These results suggest that *circMACF1* might inhibit ischemic damage and improve cardiac function after MI by repressing cardiomyocyte apoptosis.

- *Circ_0001206*

*Circ_0001206* expression is decreased significantly in H9C2 treated with H/R. The overexpression of *circ_0001206*, a sponge of *miR-665*, upregulates the expression of CRK-like proto-oncogene adaptor protein (CRKL) [75]. *Circ_0001206* regulates the *miR-665*/CRKL axis, promotes cell viability, and inhibits cardiomyocyte apoptosis.

### 5.2. The Role of Circular RNAs in Inflammatory Infiltration and MI

Cardiac cell death is often accompanied by inflammatory infiltration, which is essential for cardiac repair and significantly impacts cardiac function after myocardial injury. The circRNAs that regulate inflammatory infiltration in MI are summarized below.

#### 5.2.1. CircRNAs Promote Inflammatory Infiltration in MI

- *CircSAMD4A*

The *circular SAMD4A (circSAMD4A)* levels are overexpressed in the acute MI and H9C2 cells of mice hearts with H/R. By downregulating *miR-138-5p*, *circSAMD4A* activates the pro-apoptotic gene *Bax* and silences the anti-apoptotic gene *Bcl-2*, thereby promoting apoptosis, and dramatically increases the levels of interleukin-1 beta (IL-1β), tumor necrosis factor (TNF)-α, and interleukin-6 (IL-6) [63].

- *CircHelz*

*CircHelz*, a new circRNA transcribed from the *zinc finger (Helz)* gene, is markedly overexpressed in the ischemic myocardium and neonatal mouse ventricular cardiomyocytes under hypoxia conditions. *CircHelz* triggers a NOD-like receptor thermal-protein-domain-associated protein 3 (NLRP3) inflammasome-mediated, pro-inflammatory response in the cardiomyocytes by disturbing *miR-133a-3p* function, which leads to cardiac damage in MI [64].

- *Circ_0060745*

*Circ_0060745* is dramatically increased in the cardiomyocytes of MI mice, mostly in the myocardial fibroblasts. The knockdown of *circ_0060745* inhibits the infiltration and migration of inflammatory cells and apoptosis of cardiomyocytes by modulating the deliverability of inflammatory cytokines, including IL-6, interleukin-12 (IL-12), IL-1β, TNF-α, and nuclear factor kappa B (NF-κB), in the myocardial fibroblasts after MI or under hypoxic conditions [65]. 

#### 5.2.2. CircRNAs Inhibit Inflammatory Infiltration in MI

*CircUBXN7* is dramatically reduced in patients and mouse models of acute MI. *MiR-622*/myeloid cell leukemia 1 (MCL1) has been identified as a novel target axis of *circUBXN7* in the cardiomyocytes. The overexpression of *circUBXN7* mitigates cardiomyocyte apoptosis and the secretion of inflammatory factors, including IL-6, TNF-α, and IL-1β, leading to an inflammatory reaction in the context of H/R damage by targeting *miR-622* and preserving MCL1 expression [76]. 

### 5.3. The Roles of Circular RNAs in Collagen Deposition and Fibrosis after MI

Collagen deposition and fibrosis play stimulating roles in the remodeling process of MI hearts. The appropriate anti-fibrosis effect is critical in preventing catastrophic outcomes such as heart failure after myocardial infarction. The following paragraphs discuss some of the circRNAs that play essential roles in cardiac fibrosis after MI.

#### 5.3.1. CircRNAs Promote Collagen Deposition and Fibrosis in the Healing Stage of MI

- *CircFASTKD1*

*CircFASTKD1* is highly expressed in human cardiac microvascular endothelial cells (HCMECs). Under hypoxic conditions, *circFASTKD1* is significantly upregulated in HCMECs. The overexpression of *circFastKD1* noticeably reduces the activity of HUVECs, including the mobility and formation of vascular structures. *CircFASTKD1* serves as an endogenous sponge of *miR-106a* in endothelial cells and inhibits the YAP signaling pathway in the course of vascular endothelial progression [66].

- *CircPAN3*

*CircPAN3* is upregulated in rat hearts subjected to MI and TGFβ1-stimulated cardiac fibroblasts (CFs). *CircPAN3* enhances the expression of Foxo3 and ATG7 via the sponging of *miR-221*, which activates the autophagy process to promote fibrosis after MI [67]. By knocking down *circPAN3*, autophagic cell death and cardiac fibrosis are suppressed.

- *CircHIPK3*

*CircHIPK3* is diffusely expressed in many diseases, such as pre-eclampsia, diabetic osteoblasts, and retinal vascular dysfunction [79]. *CircHIPK3* is significantly increased in infarct hearts and hypoxia-pretreated cardiomyocytes. Upregulated *circHIPK3* can aggravate myocardial I/R damage by absorbing *miR-124-3p* [68]. Exosomal *circHIPK3* excreted by hypoxic cardiomyocytes influences the oxidative injury of cardiac microvascular endothelial cells via the *miR-29a*/IGF-1 axis [69]. Research has shown that the silencing of *circHIPK3* can reduce collagen deposition in the infarct area and improve cardiac function. *CircHIPK3* serves as a ceRNA that sponges *miR-93-5p*, thereby encouraging the excitation of the Rac1/PI3K/AKT pathway [80].

- *CircPostn*

At first, *circRNAs Postn (circPostn)* was investigated in regard to its function in cancer progression [70]. In 2020, *circPostn* was further demonstrated to be significantly increased in the plasma of MI patients, MI mouse hearts, and H/R-induced human cardiomyocytes. A deficiency in *circPostn* decreases the expression of collagen 1α1 and collagen 3α1, which inactivates the atrial natriuretic peptide (ANP) and brain natriuretic peptide (BNP) in the ventricular tissues, reduces the infarct size, and significantly alleviates myocardial injury in mice affected by MI [71]. 

#### 5.3.2. CircRNAs Suppress Collagen Deposition and Fibrosis in the Healing Stage of MI

The expression of *circNFIB* is reduced in post-MI heart tissues, TGF-β-treated NIH/3T3 cell lines, and primary adult cardiac fibroblasts. The overexpression of *circNFIB* can resolve the pro-fibrotic effects of antizyme inhibitor 1(AZIN1), c-Jun N-terminal kinase 1 (JNK1), and the transforming growth factor-β (TGF-β) SMAD family member 3 (Smad3) signaling pathway by competitively inhibiting *miR-433* [77].

Compared with the circRNAs, which promote MI, current studies on the circ-RNAs which suppress MI damage are still limited, indicating that more cardiac protective circRNAs and their underlying mechanisms must be identified in the future.

## 6. Circular RNAs as Potential Diagnostic Markers of Myocardial Infarction

When MI occurs, the occlusion of the coronary artery over a long time can lead to myocardial necrosis, eventually leading to cardiac insufficiency, malignant arrhythmias, and, indeed, sudden cardiac death. The development of urgent percutaneous coronary intervention and speed coronary artery bypass grafting has effectively decreased the mortality of patients with myocardial infarction [81]. Nevertheless, the early diagnosis and prediction of potential future MI may help to prevent or reduce the severe consequences of the disease.

The typical clinical manifestation of MI is chest pain, which also occurs in acute pericarditis and aortic dissection. Thus far, invasive coronary angiography has usually been adopted as the gold criterion when diagnosing MI. Typical ECG manifestations and blood biomarkers such as CK-MB and cTnT are key indicators for the diagnosis of MI [82,83]. Serum CK-MB begins to rise at 4 to 9 h after onset, which is too late for acute MI [84]. Some patients with hyperacute MI can present without typical ECG changes and troponin elevation [85]. This kind of patient is easily overlooked during diagnosis in the clinic. In particular, the elderly often cannot accurately describe the symptoms of chest pain. If a patient has an atypical ECG with no significant or a slight elevation of troponin, it is difficult to differentiate MI without CT coronary angiography. Early diagnosis is crucial for improving survival among these patients [86]. In addition, inadequate conditions in some hospitals can limit clinicians’ judgment of the severity of coronary artery stenosis. Therefore, there is an urgent need for a new, stable biomarker that can be used to diagnose MI. If a new biomarker that can not only diagnose MI in the early stage but also reflect the severity of coronary artery stenosis is identified in the future, this will foster convenience in the diagnosis and treatment of patients.

CircRNAs are steady and highly maintainable in different species and are significant biomarkers for the analysis of diseases [87]. They are extraordinarily stable, because the ribonuclease enzymes do not easily degrade the unexposed ends. Intriguingly, circRNAs have been observed in the bloodstream, which raises the possibility that they might enable the formation of a novel biomarker library [88]. The initial research on circRNA and MI indicated that *MI-related circRNA (MICRA)* in the peripheral blood could forecast the left ventricular function of patients with MI. Vausort et al. [89] identified *circRNA MICRA* connected with MI in peripheral blood and discovered that low *MICRA* levels are typical of MI and deteriorate left heart function. Cuimei Zhao et al. [90] employed a circRNA array to detect the expression level of circRNA in coronary blood samples from MI patients and normal subjects. In the blood of the MI patients, *Hsa_circRNA_001654* and *hsa_circRNA_091761* were significantly increased, as observed in differential expression analyses, enabling them to act as a potential biomarker for the early diagnosis of MI. Lin et al. [91] employed chip technology to identify the expression of circRNAs in the peripheral blood of acute MI patients and ordinary subjects and obtained a total of 266 diversely expressed circRNAs, of which 121 were upregulated and 145 were downregulated. The abovementioned studies indicate that the variously expressed circRNAs may be markers of the genesis and progression of MI.

In addition, peripheral blood is easier to collect, and coupled with the stability of circRNAs, this means that circRNAs in the peripheral blood are likely to contribute to the diagnosis and prognosis of myocardial infarction. Therefore, we suspect that circRNAs are a viable biomarker of MI. However, the potential role of circRNAs as a biomarker requires more specific knowledge in order to be verified. 

## 7. Perspectives

MI is a severe human health burden worldwide. However, to improve the diagnosis and treatment of MI patients and actively improve their cardiac prognosis, we must confront immense challenges in the early diagnosis and filtration of MI biomarkers. 

CircRNAs function by modulating gene expression, protein translation, and production. CircRNAs also have the features of universality, conservation, tissue features, and firmness, providing them with significant capabilities as markers of disease filtration and therapies. Due to the fast progress in high-throughput sequencing and molecular bioinformatics, circRNAs could become a novel type of disease biomarker in the future. CircRNAs have been demonstrated to be pivotal regulators of the pathogenesis of MI, and their anomalous expressions can clearly influence disease development [27]. In the treatment of cardiac hypertrophy, research teams have proactively designed artificial circRNA sponges to target *miR-132* and *miR-212*. Studies have shown that *miR-132* and *miR-212* promote cardiac hypertrophy. At the same time, circRNAs have been shown to competitively inhibit *miR-132* and *miR-212* activity and exhibit better stability than linear sponges [92]. The synthesis of MI-related circRNAs is expected to improve the survival rate of MI patients. Some circRNAs regulate MI through multiple signaling pathways, and abundant circRNAs have distinct expression levels in the plasma, tissues, and cardiomyocytes of MI patients. Due to the progress in high-throughput sequencing technology and bioinformatics, researchers can identify the circRNAs associated with MI more rapidly and easily, further revealing the molecular mechanisms that intercede in the progression of MI. The mystery of circRNAs will be revealed, offering greater insights into, and enabling the timely prevention, innovative treatment, and accurate prognosis of, myocardial infarction.

CircRNAs also play essential roles in cardiovascular disorders such as cardiac hypertrophy, atherosclerosis, and arrhythmias. Studies have reported that circRNAs are associated with cardiovascular diseases. For example, *heart-related circRNA (HRCR)* inhibits cardiac hypertrophy and heart failure by adsorbing miR-233 [93], while *circ-Foxo3* facilitates cardiac aging [94] and *circ-ANRIL* is concerned with atherosclerosis [95]. Moreover, *circRNA-284* can act as a biomarker of carotid plaque rupture [96].

Currently, there is no unified naming system for circRNAs, and standardized methods for detecting the levels of circRNAs in body fluids and tissues are required. In addition, the countless biological roles of circRNAs must be explored, and the particular mechanisms concerning various diseases remain unclear and must be clarified. This suggests that there is still much work to be done with respect to circRNAs.

## 8. Conclusions

We discussed the biogenesis, cyclization forms, and proven biological functions of circular RNAs. We focused on the positive and negative regulation of circular RNAs in myocardial infarction, primarily referring to cell death, inflammatory infiltration, and fibrosis. Moreover, this review emphasized circular RNAs as new potential biomarkers of myocardial infarction. Our review contributes to a comprehensive understanding of circular RNAs, providing a new perspective for understanding the mechanism of myocardial infarction and novel targets for the prevention and treatment of MI. In addition, the review provides references and directions for subsequent studies of the effects of circular RNAs on cardiac diseases such as myocardial infarction, cardiac hypertrophy, atherosclerosis, and arrhythmias.

## Figures and Tables

**Table 1:** Specific and multiple roles of circular RNA in the pathogenesis of myocardial infarction, including cardiomyocyte death, inflammatory infiltration, and fibrosis. They are described in detail in the text.

Name	Expression	Function	Ref.
*CircNfix*	Upregulated Downregulated	Pro-apoptotic; inhibits angiogenesis	[45,46]
*CircACAP2*	Upregulated	Pro-apoptotic	[47,48]
*CircMFACR*	Upregulated	Pro-apoptotic	[49,50]
*CircPVT1*	Upregulated	Pro-apoptotic	[51]
*CircNCX1*	Upregulated	Pro-apoptotic	[52]
*CircJARID2*	Upregulated	Pro-apoptotic	[53]
*CircROBO2*	Upregulated	Pro-apoptotic	[54]
*CircRbms1*	Upregulated	Pro-apoptotic	[55,56]
*Circ-CBFB*	Upregulated	Pro-apoptotic	[57]
*Circ-TRRAP*	Upregulated	Pro-apoptotic	[58]
*Circ-010567*	Upregulated	Pro-apoptotic	[59]
*Circ-101237*	Upregulated	Pro-apoptotic; promotes autophagy	[60]
*Circ-0124644*	Upregulated	Pro-apoptotic	[61]
*Circ-0068655*	Upregulated	Pro-apoptotic	[62]
*CircSAMD4A*	Upregulated	Pro-inflammatory infiltration; pro-apoptotic	[63]
*CircHelz*	Upregulated	Pro-inflammatory infiltration	[64]
*Circ_0060745*	Upregulated	Pro-inflammatory infiltration; pro-apoptotic	[65]
*CircFASTKD1*	Upregulated	Inhibits angiogenesis	[66]
*CircPAN3*	Upregulated	Promotes autophagy; promotes fibrosis	[67]
*CircHIPK3*	Upregulated	Promotes collagen deposition; promotes fibrosis	[68,69]
*CircPOSTN*	Upregulated	Promotes collagen deposition; promotes fibrosis	[70,71]
*CircCNEACR*	Downregulated	Inhibits programmed necrosis	[20]
*CircSamd4*	Downregulated	Inhibits apoptosis; inhibits fibrosis	[21]
*CircACR*	Downregulated	Inhibits autophagy	[42]
*CircCDYL*	Downregulated	Inducing myocardial regeneration	[72]
*CircFndc3b*	Downregulated	Inhibits apoptosis; promotes angiogenesis	[41]
*CircSNRK*	Downregulated	Inhibits apoptosis; promotes angiogenesis	[73]
*CircMACF1*	Downregulated	Inhibits apoptosis	[74]
*Circ_0001206*	Downregulated	Inhibits apoptosis	[75]
*CircUBXN7*	Downregulated	Inhibits apoptosis; inhibits inflammatory infiltration	[76]
*CircNFIB*	Downregulated	Inhibits fibrosis	[77]

## Data Availability

Not applicable.
